# A Comparative Study of Injury Severity Scales as Predictors of Mortality in Trauma Patients: Which Scale Is the Best?

**DOI:** 10.29252/beat-080105

**Published:** 2020-01

**Authors:** Mahnaz Yadollahi, Ali Kashkooe, Reza Rezaiee, Kazem Jamali, Mohammad Hadi Niakan

**Affiliations:** 1 *Trauma Research Center, Shiraz University of Medical Sciences, Shiraz, Iran*; 2 *Student Research Committee, Shiraz University of Medical Sciences, Shiraz, Iran*

**Keywords:** Trauma Injury Severity Score, Revised Trauma Score, Injury Severity Score, Mortality, Injury

## Abstract

**Objective:**

To compare the injury severity scales as predictors of mortality in trauma patients to search for the best scale.

**Methods:**

In a prospective cohort study and systematical random sampling conducted from March to September 2017, trauma patients over the age of 13 years were enrolled. The investigated variables were age, gender, systolic blood pressure, heart rate, respiratory rate, injured body region, Glasgow Coma Scale (GCS), injury severity score (ISS), revised trauma score (RTS), trauma injury severity score (TRISS) and the outcome.

**Results:**

Totally, 1410 trauma patients were followed up, out of which 68.5% were male. The participants’ mean age was 43.5±20.88 years. After adjusting the confounding effects, age over 60 years (OR=7.38, CI [3.91-13.93]), GCS<8 (OR=6.5, CI [2.38-18.16]), RTS<7.6 (OR=6.04, CI [2-13.7]), and TRISS<0.9 (OR=3.09, CI [1.39-6.88]) were determined as the most significant predictor variables for in-hospital mortality. The results of Receiver Operating Characteristic (ROC) curve revealed that TRISS had the highest area under the curve in comparison to other tests that were evaluated. Furthermore, TRISS had the highest sensitivity and specificity for scores higher than 96.15. By contrast, the sensitivity and specificity of GCS decreased for scores higher than 5.5.

**Conclusion:**

Our results showed that TRISS, RTS, GCS, and ISS were all very effective approaches for evaluating prognosis, mortality and probable complications in trauma patients; thus, these systems of injury evaluation and scoring are recommended to facilitate treatment. TRISS, RTS, and ISS had almost the same sensitivity that was higher than GCS, but GCS had the most specificity. Finally, TRISS was selected as the most efficient scale for predicting mortality.

## Introduction

Trauma is one of the biggest issues in healthcare system and a major cause of mortality worldwide [1]. It is the fourth leading cause of death all around the world and the leading cause of death and disability in developing countries [2-4]. Assessments regarding mortality due to trauma in 2003 revealed a rate of 99 deaths per 100,000 populations worldwide, and this rate was 58 deaths per 100,000 populations in Iran [5]. What’s more; trauma, whether directly or indirectly, burdens the society with heavy economic and social costs [6, 7]. 

Hence, it has prompted the healthcare policy-makers and officials to take fundamental measures in this regard [6, 7]. Deaths due to trauma have a high portion of all fatalities occurring in a society. Convenient access to hospitals and improved facilities of emergency medical transportation would allow patients to be transferred to hospitals before dying; meanwhile, in-hospital deaths have a close association with the hospital’s facilities, type of hospital services, quality of medical and nursing services, and finally injury severity assessments [8].

The main factor in assessing injury severity is the type of scoring system under use or injury severity grading, which is considered as a basic requirement for trauma management and clinical tests [9]. The observed statistical differences in the rate of long-term disabilities following trauma between different healthcare centers can illustrate the differences in injury severity grading and quality of patients’ management in each population under study. In other words, for an accurate management of the traumatic patients, it is essential to have a suitable tool or index for their evaluation. 

Trauma Injury Severity Score (TRISS), Revised Trauma Score (RTS), Injury Severity Score (ISS) and Glasgow Coma Score (GCS) are a few examples of such trauma indices [10]. Different studies have reported various findings using trauma indices. In 2011, Kondo *et al*. conducted a study in Japan to evaluate the trauma scoring systems. Their final results showed that GCS, age and systolic blood pressure, which represent the RTS system, can be used for proper assessment of patients and in-hospital deaths [11]. 

Moreover, this system could effectively predict the patient survival probability and help the physicians to make more accurate medical choices [11]. In the meantime, Hariharan *et al*. did a study on 326 trauma patients in Trinidad, which confirmed the high value and efficiency of TRISS in prognosis evaluation, and introduced the index as a standard scoring system [12]. Considering that each of these scoring systems can play an important role in estimating mortality rates in trauma patients, the present study aimed to evaluate the injury severity scales and the predictors of trauma related mortalities as well as to discover the scale of choice for traumatic patients.

## Materials and Methods

*Setting*

This prospective cohort study was conducted in Rajaee Hospital, the main referral trauma center of emergency medical services in Fars Province, southwestern Iran. This center has 7 general wards each comprising of 32 beds, 2 emergency wards with 20 beds each, and 6 intensive care units each providing 9 beds. In addition, the study protocol was approved by the institutional Ethics Review Board affiliated to Shiraz University of Medical Sciences, Shiraz, Iran. 

*Data collection*

The present study was conducted on injured patients who had referred to the hospital’s emergency department from March to September 2017. After being screened by the emergency physicians, each patient was given a unique 8-digit code assigned by the admission unit. Trauma patients over 13-years-old who were under supervision and treatment in the emergency room for at least 6 hours were included in this study. Outpatients, patients who were under treatment for less than 6 hours, and patients younger than 13-years- old, those who had expired upon arrival, and those with previous history of disease (cardiovascular, pulmonary, renal, or cerebral, such as stroke) were excluded. 

Patients younger than 13-years-old were excluded, because this center only admits patients older than 13 years. Moreover, patients who were admitted for less than 6 hours were omitted due to less severity of injury in this group of patients. During the study period, a total of 14,100 patients were admitted to the hospital and evaluated based on the inclusion and exclusion criteria. Then, one out of each 10 patients was assigned a number through systematic randomization and selected to form our sample size. 

A cluster sampling was used to select the samples from the community. The probability of sample selection was the same for all patients. Therefore, the severity of injury had a normal distribution from the community. Finally, 1,410 patients entered the study and were followed up by trained individuals from the moment of admission to the time of discharge or death in hospital; based on the study’s inclusion and exclusion criteria. Data collection was done using checklist containing demographic information, injured body regions, clinical findings at the time of admission, vital signs, and trauma indices (GCS, ISS, TRISS, and RTS). The mentioned variables as well as follow up of the patients and their survival made up our variables.

*Definitions*

In this study, the objective (GCS), physiologic (RTS), anatomic (ISS), and anatomic-physiologic (TRISS) traumatic injury severity scales were estimated based on the following criteria: The GCS was composed of eye, verbal, and motor components, which received 4, 5, and 6 points, respectively (total of 15 points) [13]. The RTS was scored from the first set of data obtained on the patient variables, and consisted of GCS, respiratory rate, and systolic blood pressure. RTS was calculated using the codes provided in the Table 1 and the formula of

RTS=0.93 GCS+0.73 SBP+0.29 RR.

**Table1:** Calculating revised trauma score

**RR**	**SBP**	**GCS**	**Code**
10-29	89<	13-15	4
29<	76-89	9-12	3
6-9	50-75	6-8	2
1-5	1-49	4-5	1
0	0	3	0

Abbreviations: GCS, Glasgow coma scale; SBP, systolic blood pressure; RR, respiratory rate

The RTS value ranges between 0-7.8408, where the lower scores indicate more severe injuries [14, 15]. To calculate the ISS value, we first determined the AIS. For this purpose, the patients’ injuries were categorized according to 6 body regions (head and neck, face, chest, abdomen, extremities, and external) based on the 2005 guidelines (AIS-2005). Each of these anatomical regions received a score ranging from zero (no lesion) to six (lethal lesion) based on injury severity [3]. 

To calculate ISS, we added the squares of the three highest AIS values (for different body regions) together. ISS values ranged between 0-75. If an injury assigned an AIS of 6, the ISS score was automatically assigned to 75 [16]. TRISS was a combined index that in addition to the ISS considered the patients’ age, trauma mechanism and vital signs. The TRISS scale had two separate subsets for adults over the age of 15 including (i) Trauma with a blunt (non-penetrating) mechanism and (ii) Trauma with a penetrating mechanism.

Furthermore, this scale also had a subset for children less than 15 years of age, in which the trauma mechanism was ignored. The coefficients in this model mostly indicated the probability of survival ($P_{S}$) rather than the probability of death ($P_{D}$); in this regard, the equation $P_{D}=1-P_{S}$naturally applies. $P_{S}$ was defined for each patient as $P_{S}$=1/(1+$e^{-b}$). For adults experiencing blunt trauma, b=-0.44+(0.80*RTS)–(0.08*ISS)–(1.74*age). For adults experiencing penetrating trauma, b=- 2.53+(0.99*RTS)–(0.06*ISS)–(1.13*age).

Age was specified through the values zero and one; zero was related to patients between 15-54 years old and one represented the patients over 55 years. We categorized the proper cut-off points for trauma indices according to previous studies [10, 17] and the vital signs according to laboratory standards. Moreover, the variable of age was divided into two categories, over 60 and under 60 years.

*Statistical analysis*

Descriptive indicators were expressed as means (± standard deviation) or percentages using the obtained data. Univariate analysis and Chi-square test were used to discover the individual relationships between each variable and mortality rate. Logistic regression with backward method was employed to determine the independent variables predicting mortality. Finally, the area under the receiver operating characteristic (ROC) curve was used to determine the efficiency of injury severity scale and to detect the sensitivity and specificity in order to predict the status of discharge “Death or Alive”. 

Normality of data distribution was assessed through the Kolmogorov-Smirnov test and in cases of non-normality, medians and quartiles were used for the reporting purposes. The produced results and respective data were analyzed via SPSS Statistics software (Version 16.0, Chicago, IL, USA). Also, *p*<0.05 was considered as significance level in all tests.

## Results

A total of 1410 trauma patients were followed up in this study, out of which 68.36% were male. The patients had a mean age of 43.5±20.88 years. Table 2 presents the univariate relationships between the parameters under study and mortality due to trauma. Furthermore, each parameter’s average was provided stratified by survivals and fatalities. Findings from the table revealed that gender was the only variable without any significant association with mortality caused by trauma. (*p*=0.16)

**Table2:** Univariate relationship between different variables and mortality in traumatic patients

**Variables**		**Survived (1279)**	**Un-survived (130)**	***P*** ** value**	
Age*		42.05±20.19	57.77±22.20		<0.001
**Gender**	**Male**	869 (67.94 %)	96 (73.85%)		0.16
	**Female**	410 (32.06 %)	34 (26.15%)		
SBP*		127.8±19.50	110.58±42.65		<0.001
HR*		83±14	95±31		<0.001
RR*		17.96±2.97	19.50±5.81		<0.001
GCS*		15±0	10±7		<0.001
**ISS**	**Mean±SD**	6.83±6.15	18.68±14.19		<0.001
	**Median±IQR**	7±5	16±16		
RTS*		7.78±0.33	5.9±1.77		<0.001
TRISS*		98.60±2.15	74.57±27.5		<0.001
**Body region**	**Head & neck**	144 (11.26%)	22 (66.92%)		<0.001
	**Face**	27 (2.11%)	0 (0.00%)		
	**Thorax**	33 (2.58%)	3 (2.31%)		
	**Abdomen**	20 (1.56%)	5 (3.85%)		
	**Vertebra**	76 (5.94%)	1 (0.77%)		
	**Extremities**	370 (28.93%)	12 (9.23%)		
	**Body surface**	2 (0.16%)	0 (0.00%)		
	**Multiple**	607 (47.46%)	87 (66.92%)		

* Mean±SD; Abbreviations: SBP, systolic blood pressure; HR, heart rate; RR, respiratory rate; GCS, Glasgow coma scale; ISS, injury severity score; RTS, revised trauma score; TRISS, trauma injury severity score.

Inter-variable relationships and odds ratios were analyzed using logistic regression with backward method; the results of this analysis were provided in Table 3. In this table adjusted OR with 95% uncertainty was presented. All variables were entered into the model, and finally the model with these specific variables had best goodness of fit. The findings revealed that systolic blood pressure was the only indicator without a significant relationship with prediction of in-hospital mortality due to trauma (*p*=0.18). After adjusting the confounding effects, age>60 years (OR=7.38 [3.91-13.94]), GCS<8 (OR=6.57, CI [2.38-18.16]), ISS>15 (OR=3.28, CI [1.54-6.98]), RTS<7.6 (OR=6.04, CI [2.00-13.07]), and TRISS<0.9 (OR=3.09, CI [1.39-6.88]) were found to be the most important predictors of in-hospital mortality.

**Table3:** Results of logistic regression analysis regarding the effects of different injury severity scales and variables on mortality rate

**Variable**		**OR** *** (95%CI)***	**P value**
**Age**	**<60**		<0.001
	**>60**	7.38 (3.91-13.94)	
**SBP**	**>120**		0.18
	**60<SBP<120**	1.07 (0.60-1.92)	
	**<60**	2.29 (0.35-15.19)	
**GCS**	**>8**		<0.001
	**<8**	6.57 (2.38-18.16)	
**ISS**	**<15**		<0.001
	**>15**	3.28 (1.54-6.99)	
**RTS**	**>7.6**		<0.001
	**<7.6**	6.04 (2.00-13.07)	
**TRISS**	**>0.9**		0.008
	**<0.9**	3.09 (1.39-6.89)	

Abbreviations: SBP, systolic blood pressure; GCS, Glasgow coma scale; ISS, injury severity score; RTS, revised trauma score; TRISS, trauma injury severity score.

To compare the efficiency of the four scoring methods, i.e. TRISS, ISS, GCS, and RTS, we made a comparison of the areas under the ROC curves for each respective index in order to analyze the level of sensitivity and specificity. Positive predictive value and negative predictive value of model for prediction of death or survival were 73.45 and 96.71, respectively. In other words, TRISS, ISS, GCS, and RTS have the ability to correctly diagnose death up to 73.45%, and also they can correctly diagnose survival up to 96.71% of cases. 

**Fig. 1:**
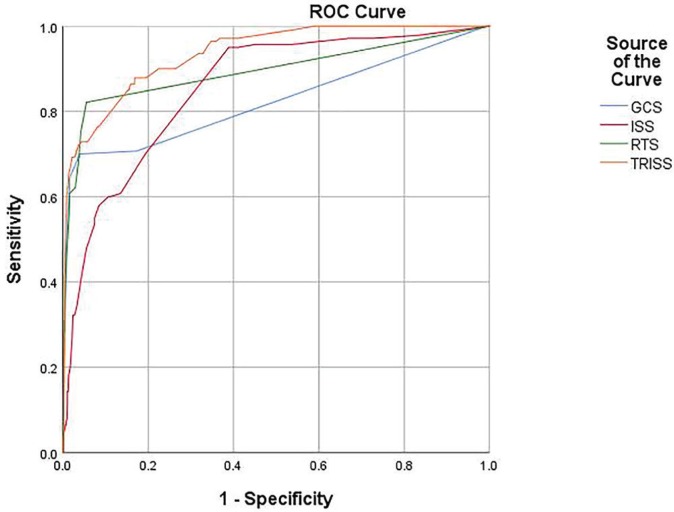
Receiver operating characteristic curve of injury severity scales (TRISS, RTS, GCS, and ISS).

The findings reveal the areas under the ROC curves as 0.93, 0.80, 0.75, and 0.85 for the trauma indices of TRISS, ISS, GCS, and RTS, respectively. Needless to say, these results indicated the higher ability of TRISS in predicting trauma outcomes (Figure 1). Furthermore, Table 4 shows the sensitivity and specificity of each system in predicting trauma outcomes. The findings regarding the ROC curve denoted to a higher level of sensitivity in TRISS and RTS. Also, TRISS had the highest sensitivity and specificity for scores higher than 96.15. The opposite is true for the GCS; as the sensitivity and specificity of this test decreasd for scores higher than 5.5. What’s more, GCS had the lowest sensitivity (67.96%) and highest specificity (82.69%) in our analysis.

**Table 4.** Analysis of area under receiver operating characteristic curve

**Injury severity scales**	***RTS***	***TRISS***	***ISS***	***GCS***
**Area under curve**	0.85	0.93	0.80	0.75
**Sensitivity (%)**	95	95	94.4	67.96
**Specificity (%)**	67	70	60.1	82.69
**Score of system with high sensitivity and specificity**	≥7.69	≥96.15	≤8.5	≤5.5

Abbreviations: ISS, injury severity score; RTS, revised trauma score; TRISS, trauma injury severity score, GCS.

## Discussion

Results from ROC curve analysis of the injury severity scores in the present study suggested that TRISS has the highest efficiency in predicting mortality and trauma outcomes. In a study on 329 trauma patients in Trinidad, TRISS was introduced as a standard system for the evaluation of prognosis in trauma patients [12]. Moreover, Moon *et al*. considered TRISS as a useful tool to evaluate trauma patients with brain injury, and introduced the method as being simple and efficient in terms of time management [18]. 

Despite the present study and other mentioned studies verifying the value and efficiency of TRISS in prognosis trauma patients, a study conducted on 300 hospitalized trauma patients in India evaluated TRISS scale and its relative coefficients, which determined that the method was not a suitable tool in predicting the probability of survival [19]. Our findings showed that following TRISS, RTS had the highest efficiency in assessing traumatic injury severity. Similar to the present study, a study of trauma patient assessment systems in 2011 found the RTS to be a suitable system for determining the prognosis in trauma cases, and their results revealed that by using the GCS, age, and systolic blood pressure that represent the RTS system, we can make a proper evaluation of patients and predict the in-hospital fatalities. 

Furthermore, this system is capable of predicting the probability of survival, which can help the physicians to make more accurate treatment choices [20]. The mean RTS scores obtained for survivals and fatalities in our research were consistent with the results from multiple studies [18, 19, 21]. This shows the high efficiency of RTS in evaluation of prognosis in trauma patients; however, TRISS is still preferable. In the present study, the evaluations based on the area under the ROC curve indicated the lower efficiency of the ISS in predicting mortality compared to TRISS and RTS. 

Sammour *et al*. also recognized TRISS as a very accurate factor (area under ROC curve: 0.96), and considered ISS to have less accuracy in comparison (area under ROC curve: 0.85), a result that is consistent with our findings [22]. What's more, our findings revealed an ISS sensitivity of 94.4% and an ISS specificity of 60.1%. Meanwhile, a study in Taiwan investigating the risk factors of mortality in elderly trauma patients arrived at an ISS sensitivity of 81.3% and an ISS specificity of 88.7% [23].

Moreover, our results showed that ISS was significant for both survivors and fatalities. Numerous studies have shown the relationship between increased ISS and increased mortality rates and complications [24, 25]. For instance, in a research conducted in Iran, age and ISS were the most important factors in mortality [26]. In addition, age was detected as a significantly important factor in predicting mortality in our survey. In another study related to geriatric trauma, the age-group of over-65 showed a higher mortality compared to younger groups despite having a lower ISS [23].

In the present study, patients with GCS<8 had higher odd ratio of mortality in comparison with the patients with GCS>8. This result is consistent with the results of another study, indicating that GCS<8 are the most significant mortality risk factors for trauma patients in the first hour following admission [27]. Beside these, in a study on 740 trauma patients, GCS<8 was believed to be an effective factor in predicting mortality [28]. Regarding the prediction of mortality in ROC curve analysis, the best cut-off point for the GCS in our study was ≤5.5, with 67.96% sensitivity and 82.69% specificity. 

A study in Northern Iran revealed that GCS can predict mortality with 98.4% sensitivity and 92.3% specificity in the scores ≤8 [29]. However, GCS was the most powerful scale in predicting mortality in the mentioned study; this scale had the lowest efficiency in predicting mortality in comparison with the other scales in our study. These discrepancies in our research with the mentioned research could have been due to the fact that their study only included the pediatric patients, in contrast with our survey that included patients more than 13 years old.

Regarding demographic variables in the population under study, the trauma patients had a mean age of 43.5±20.88 years. Meanwhile, similar studies reported a mean age around 30 years [30, 31]. Moreover, our study asserted that trauma and its consequent mortality mainly occur in men, which was consistent with the results obtained from numerous trauma-related studies, showing the majority of injured and deceased patients to be males [27, 32-34]. Another issue to remember is that multiple organ traumas had the highest frequency in our study and most fatality cases had also experienced multiple organ injuries. 

In a study related to geriatric trauma, most in-hospital deaths had occurred in patients with an ISS>16 and individuals with severe injuries to the brain, thorax, abdomen, and pelvis had the highest mortality rate compared to other regions [35]. The present study had several strong points as follows. The large number of patients under study, evaluation of different variables and a follow up period up to the time of discharge in a trauma referral center in southern Iran. Despite the significance of this issue, there are few related studies in Iran; and naturally, our study expanded the literature in this domain. 

On the other hand, the present study had some limitations, such as not including traumatic patients younger than 13 years and pediatric patients and those with higher ISS. Too much time was spent to calculate ISS, and the assignment of injury code to patients with damages was a complex process. For future research, we suggest the comparison of trauma scoring methods in terms of prognosis in various age-groups, including the elderly and pediatric population. Our findings showed TRISS, RTS, GCS, and ISS as effective methods for evaluation of prognosis, mortality and probable complications in trauma patients. Therefore, these systems of injury evaluation and scoring are recommended for facilitation of treatment. Furthermore, TRISS, RTS, and ISS had almost the same sensitivity, which was higher than GCS. On the other hand, GCS had the most specificity. Finally, TRISS was selected as the most efficient scale for predicting mortality.

## Funding/Support:

The study was financially supported by Shiraz University of Medical Sciences as a thesis project supervised by Dr. Mahnaz Yadollahi and Dr. Kazem Jamali.
